# Development of a Competitive Enzyme-Linked Immunosorbent Assay Based on Purified Recombinant Viral Protein 7 for Serological Diagnosis of Epizootic Haemorrhagic Disease in Camels

**DOI:** 10.1155/2022/5210771

**Published:** 2022-03-21

**Authors:** Anna Serroni, Simonetta Ulisse, Mariangela Iorio, Caterina Laguardia, Lilia Testa, Gisella Armillotta, Marco Caporale, Romolo Salini, Davide Lelli, Ulrich Wernery, Rekha Raghavan, Maria Teresa Mercante, Mauro Di Ventura

**Affiliations:** ^1^Istituto Zooprofilattico Sperimentale dell'Abruzzo e del Molise G. Caporale, Teramo, Italy; ^2^Istituto Zooprofilattico Sperimentale della Lombardia e dell'Emilia Romagna Bruno Ubertini, Brescia, Italy; ^3^Central Veterinary Research Laboratory, Dubai, UAE

## Abstract

Epizootic haemorrhagic disease virus (EHDV) is a member of the *Orbivirus* genus in the Reoviridae family, and it is the etiological agent of an arthropod-transmitted disease that affects domestic and wild ruminants. Due to its significant economic impact, many attempts have been done in order to develop diagnostic immunoassays mainly based on the use of the viral protein 7 (VP7), that is, the immunodominant serogroup-specific antigen. In this work, a recombinant VP7 (recVP7) of EHDV serotype 2 was produced in a baculovirus system, and after purification using ion metal affinity chromatography, we obtained a high yield of recombinant protein characterized by a high degree of purity. We used the purified recVP7 as reagent to develop a competitive enzyme-linked immunoassay (c-ELISA), and we tested the presence of EHDV antibodies in 185 dromedary camel serum samples. The c-ELISA showed good performance parameters in recognising positive sera of naturally EHDV-infected dromedary camels; in particular, our developed test reached 85.7% of sensitivity, 98.1% of specificity, 93% of accuracy, and a high agreement value with results obtained by the commercial ELISA kit (Cohen's kappa value of 0.85) that we adopted as the reference method. This c-ELISA could be a useful screening test to monitor the virus spread in camels that are sentinel animals for endemic areas of disease.

## 1. Introduction

Epizootic haemorrhagic disease (EHD) is an important arthropod-transmitted disease that mainly affects white-tailed deer (*Odocoileus virginianus*) and cattle [1].

The causative virus, Epizootic haemorrhagic disease virus (EHDV), is widespread among wild cervids in North America, where the disease is endemic and periodically causes serious epidemics [2, 3]. It is responsible of occasional deaths of wild animals such as bisons and elks [4, 5] and in the last decade caused outbreaks in cattle and other ruminants in America, Africa, Asia, Australia, the Middle East, and some islands of the Indian Ocean [6–12]. In 2006, EHDV was identified as the causative agent of a bluetongue-like disease in Tunisia, Algeria, and Morocco [12–14]. The peracute form, primarily characterized by fever, anorexia, and respiratory distress, usually affects white-tailed deer, with high mortality rate [1, 15]. Less severe clinical signs are described for EHDV-infected cattle [16], including also asymptomatic infection [8]. Different studies reported that sheep, goats, and camels seem susceptible to EHDV infection despite showing no clinical signs; however, their epidemiological role in the transmission of the disease is yet to be clarified [1, 15, 17, 18].

Epizootic haemorrhagic disease virus is a double-stranded RNA virus that belongs to the *Orbivirus* genus in the Reoviridae family, composed of ten double-stranded RNA segments that encode seven structural proteins (VP1–VP7) and five nonstructural proteins [1, 19–22].

Among the structural proteins, VP2 and VP5 form the outer-capsid layer and their function is related to the ability of the virus to bind to the cell receptor during infection and to the mechanism of host cells penetration [9].

To date, the variable region of VP2 allows the recognition of at least seven EHDV serotypes. Two additional new putative serotypes have been recently suggested but, at the moment, they are not included in the official panel of the virus reference strains [1, 15, 21, 23, 24].

The structural proteins VP7 and VP3 are part of the inner capsid, the first forming a bilayered icosahedral core particle, and the second, in the subcore, surrounding the proteins involved in the viral transcriptional process together with dsRNA genomic segments [9, 25]. VP7 is the most abundant *Orbivirus* structural protein, with a molecular weight of 40 kDa, and is the main immunodominant antigen [26–28]. It is highly conserved within each *Orbivirus* species; therefore, it represents an efficient target for group-specific serological diagnosis [25, 29]. EHDV is closely related to bluetongue virus, and this similarity had made problematic the development and the selection of serological diagnostic tests because of the difficulty to avoid cross-reactivity [15]. To date, competitive ELISA, based on the use of monoclonal antibodies that recognise EHDV VP7, represents the preferred technique to detect antibodies against EHDV.

In this work, we describe a baculovirus-based method for the production and purification of the recombinant VP7 of EHDV serotype 2. The purified protein was used to develop a c-ELISA for detecting antibodies directed against EHDV VP7. The performance of the method in recognising positive sera of naturally EHDV-infected camels was evaluated.

## 2. Materials and Methods

### 2.1. Cell Lines and Baculovirus Stock

Sf9 insect cells (ECACC 05011001) derived from pupal ovarian tissue of the fall armyworm, *Spodoptera frugiperda*, were used. The cells were maintained at 27°C in 0.2 *µ*m vent cap Erlenmeyer flasks (Corning) and made to grow in suspension with Sf900II serum-free medium (Gibco, Cat# 10902088) in a shaker incubator at 110 rpm rotation.

A recombinant baculovirus bearing EHDV VP7 gene was synthesized commercially from GenScript (Piscataway, New Jersey, USA).

To amplify the viral stock, Sf9 insect cells were seeded in a shaker flask at a density of 1.5 × 10^6^ cells/mL and infected with P2 at a multiplicity of infection (MOI) of 0.01. The cells were placed in a shaker incubator at 27°C at 110 rpm, and the cell density, viability, and diameter were monitored at 24, 48, and 72 h postinfection (p.i.). The viral titres at the same three collection times were also evaluated. To obtain the P3 stock, cell supernatant was harvested at 72 h p.i., by centrifugation at 2700×*g* for 10 min, immediately stored at 4°C, supplemented with 10% of dimethyl sulfoxide (DMSO) and the aliquots stored at −80°C. The titres of P2 and P3 viral stocks were evaluated using the cell line Sf9 Easy Titre (Sf9 ET), based on the end-point dilution assay [30]. The Sf9 ET cell line had been stably transfected with plasmid DNA containing the enhanced green fluorescent protein (eGFP) gene under the control of the baculovirus polyhedrin promoter. Therefore, the cells turn green when infected with baculovirus due to activation of the polyhedrin promoter/eGFP complex. Uninfected Sf9 ET cells do not express detectable GFP.

Briefly, a suspension of Sf9 ET cells at a density of 8 × 10^5^ cells/mL was added in a 96-well microplate, and an end-point dilution of the virus stock was prepared and inoculated onto the Sf9 ET cell culture. After five days, the microplate was observed using fluorescence microscopy, and baculovirus stock titres were calculated according to the Reed e Müench method [31]. The result was obtained in TCID_50_/mL (median tissue culture infectious dose), that is multiplied by the conversion factor 0.7, giving the corresponding value in PFU/mL.

### 2.2. Production of EHDV recVP7 Protein

In order to find the best conditions for the recombinant protein expression, small-scale productions have been carried out. In particular, the EHDV recVP7 expression level was checked at different times of harvest (TOH) of Sf9 cells infected with P3 baculovirus at different MOIs. Briefly, recVP7_EHDV P3 viral stock was used at MOI 0.001, 0.01, and 0.1 to infect 50 mL of Sf9 cell suspension with a density of 3.5 × 10^6^ cells/mL. Cell viability and cell diameter were evaluated using the Countess automated cell counter (Thermo Fisher Scientific) at different timepoints postinfection (0, 24, 48, 72, 96, and 120 h p.i.). At each time of harvest and for each MOI used, the titres were evaluated using Sf9 ET cell culture as above described.

For large-scale production, 1500 mL of Sf9 cells at a density of 3.5 × 10^6^ cells/mL and viability not less than 98% were infected with a P3 baculovirus at 0.01 MOI, incubated at 27°C, and kept in suspension with shaking at 110 rpm. The infected cells expressing the recombinant protein were harvested at 72 h p.i. by centrifugation at 2700×*g* for 10 min.

The cell pellet was washed once with phosphate buffered saline pH 7.5 (PBS) and stored at −20°C until purification. The cell supernatant was immediately supplemented with 0.2 M L-arginine hydrochloride and incubated by mild magnetic stirring for 1 h at room temperature (RT), and subsequently, it was placed at 4°C overnight until the purification by immobilized metal affinity chromatography (IMAC).

### 2.3. Purification of recVP7 EHDV Protein from Pellet

The pellet previously washed and stored at −20°C was thawed and resuspended with lysis buffer (10 mM Tris-HCl, pH 7.5, 0.5% NP-40, 3.3 M L-arginine hydrochloride) and supplemented with protease inhibitors cocktail according to the manufacturer instructions (Roche, #Cat 11873580001).

The resuspended pellet was placed on ice in mild agitation, at 4°C, for 3 h, and then centrifuged at 2700 × *g* for 10 min. The pellet was discarded, and the supernatant was recovered and treated according to the procedure described by Luo and Sabara [32], with minor modifications. Briefly, 25 mL of a 4 M saturated ammonium sulphate solution, prepared in 100 mM Tris-HCl buffer, pH 7.5, was added for every 100 mL of supernatant. To facilitate the protein precipitation, the solution was incubated at 4°C with gentle agitation overnight (o.n.). The precipitated proteins were collected by centrifugation at 16000 × *g* for 10 min.

The supernatant was discarded, the pellet was resuspended in 10 mM Tris-HCl buffer, pH 7.5, and centrifuged for 10 min at 16000×*g*. The supernatant was collected and stored at 4°C until purification by IMAC.

### 2.4. Purification of recVP7 from Pellet and Supernatant by Ion Metal Affinity Chromatography (IMAC)

Sf9 pellet treated as previously described and Sf9 supernatant both containing the EHDV recombinant VP7 were subjected to IMAC, according to the method described by Ulisse et al. [33].

The purification was conducted in a fully automated manner using an AKTApurifier 100 instrument according to the manufacturer instructions. Briefly, the HisTrap excel column (Cytiva, #Cat 17371206) was equilibrated with the equilibration buffer (20 mM sodium phosphate, 500 mM sodium chloride, and 0.2 M L-Arginine hydrochloride). Before loading to the column, the recVP7 recovered from Sf9 pellet was diluted in 350 mL of equilibration buffer to avoid potential clogging of the column. The cell supernatant, instead, was directly loaded onto the column without any treatment. After loading separately recVP7 from pellet and from supernatant, the column was washed with 20 mM imidazole in equilibration buffer and the recombinant proteins were finally eluted with 20 mM sodium phosphate, 500 mM sodium chloride, 0.2 M L-arginine hydrochloride, and 250 mM imidazole.

Purified recVP7 were stored at 4°C o.n. and centrifuged at 4000 × *g* using Amicon Ultra-15 Centrifugal Filter Units MWCO 10 kDa (Merck Millipore, #Cat UFC901024). The concentrated recVP7 samples were diluted with PBS, pH 7.5, and were supplemented with 0.5% sarkosyl. Bradford assay was performed to assess the concentration of purified recVP7 that was obtained, respectively, from pellet and from supernatant. The purified protein was finally stored at 4°C until characterization by sodium dodecyl sulphate polyacrylamide gel electrophoresis (SDS-PAGE) and Western blotting.

### 2.5. SDS-PAGE and Western Blotting

Recombinant VP7 purified from Sf9 cell pellet and supernatant were checked for purity by SDS-PAGE and characterized by Western blotting using the anti-VP7 mAb, clone 4G11 (IZSLER) raised against semipurified EHDV field strain serotype 7 obtained from Kimron Institute (Israel) and the anti-V5 antibody (Thermo Fisher Scientific, Cat#R961-25, RRID: AB_2556565) both conjugated with HRP. Uninfected Sf9 cells were used as negative control.

Briefly, the same quantity of purified protein derived from cell pellet and supernatant was denatured for 10 min at 70°C, as suggested by Thermo Fisher Scientific instructions, separated on NuPAGE Novex 4–12% Bis-Tris Gels (Thermo Fisher Scientific, #CatNP0321BOX) and stained with Biosafe Coomassie G-250 stain (Bio-Rad, #Cat 1610786).

Recombinant VP7 samples fractionated by electrophoresis were also transferred onto nitrocellulose membranes and tested for immunoreaction, incubating the membranes with the anti-VP7 mAb clone 4G11 (IZSLER) and the anti-V5 antibody at 1 : 30000 dilution and 1 : 10000 dilution, respectively, both conjugated with HRP, at 4°C overnight. After rinsing membranes with PBST, the Amersham ECL Select Western Blotting Detection Reagent (Cytiva, Cat# RPN2235) was used to acquire images of EHDV recVP7.

### 2.6. i-ELISA

Both proteins purified from the infected Sf9 cell pellet and the infected Sf9 cell supernatant were used as antigen and tested with different dilutions of an EHDV-positive bovine serum (IZSAM) using indirect ELISA (i-ELISA).

Three different protein lots were tested. The recombinant proteins from pellet and supernatant (pVP7 and sVP7, respectively), were coated on 96-well MaxiSorp microplates (Thermo Fisher Scientific, Cat# 44-2404-21) at 2 µg/mL in 0.05 M carbonate-bicarbonate buffer (pH 9.6) with a volume of 100 *µ*L/well and incubated at 4°C o.n.

After incubation, the 96-well microplates were washed once with PBS, pH 7.5, containing 0.05% Tween-20 (VWR, Cat# 7374.1000) (PBST), saturated with 200 *µ*L/well using 1% (w/v) skimmed milk in PBST (blocking buffer) and incubated for 1 h at 37°C.

The 96-well microplates were washed three times again with PBST, and the positive and the negative sera (50 *µ*L/well) at different dilutions (1 : 50, 1 : 100, and 1 : 200) were added. The microplates were incubated for 1 h at 37°C, and after further three washes with PBST, the HRP-conjugated antibovine mAb (1.5 mg/mL) at 1 : 20000 dilution (50 *µ*L/well) (Merck Cat# SAB3700020) was added.

After 1 h at 37°C, the 96-well microplates were washed three times with PBST, 3,3′,5,5′-tetramethyl benzidine substrate (TMB) (Surmodics, Eden Prairie, USA, Cat# TMBW-0060-01-3) was added (100 *µ*L/well), and the microplates were incubated at RT for 30 min.

The reaction was stopped by adding 0.5 N sulphuric acid (50 *µ*L/well), and the optical density at 450 nm (O.D. 450) was measured.

### 2.7. c-ELISA

Competitive ELISA was performed using recombinant sVP7 as antigen and anti-VP7 mAb clone 4G11 (IZSLER) as the detector system to measure the competition generated by test sera for binding to the viral protein to test a total number of 185 dromedary camel sera provided by the Central Veterinary Research Laboratory, Dubai, UAE.

Briefly, the microplates were coated with sVP7 at 2 *µ*g/mL in 0.05 M carbonate-bicarbonate buffer, pH 9.6 (100 *μ*L/well), saturated and washed as above described. The undiluted dromedary camel sera, the positive and negative bovine sera (IZSAM reference sera), used as controls, were added (50 *µ*L/well), and the microplates were incubated at 37°C for 1 h. The microplates were washed three times with PBST, and the HRP-conjugated anti-VP7 mAb clone 4G11 (10 mg/mL, IZSLER) at 1 : 20000 dilution in blocking buffer (50 *µ*L/well) was added. After 1 h at 37°C, the microplates were further washed three times with PBST, 3′,5,5′-tetramethyl benzidine substrate (TMB) (Surmodics, Eden Prairie, USA, Cat# TMBW-0060-01-3) was added, and the microplates were incubated at RT for 30 min. The reaction was stopped by adding 0.5 N sulphuric acid, and the O.D. 450 nm was measured. The same protocol was performed to evaluate the analytical specificity using a total of 55 AHSV-positive sera and BTV-positive sera.

All the c-ELISA data were normalized using the following formula: (O.D._450nm_ serum sample value/O.D._450nm_ mAb value) × 100.

All the sera samples were tested using the commercial kit ID Screen EHDV Competition (IDVet, Grabels, France, Cat# EHDVC5P) according to manufacturer instructions to assess positive and negative samples and compare the results of the two methods.

The results were elaborated using R Core Team software [35]. The receiver operating characteristic (ROC) curve obtained was used to define the test cutoff value. The sensitivity, specificity, and accuracy values of the method were also calculated. The Cohen's kappa value was calculated to determine the agreement between the new methods and the commercial kit.

## 3. Results

### 3.1. Recombinant VP7 Production

Baculovirus P2 viral stock expressing EHDV recVP7 was used for P3 viral stock production. The P3 baculovirus stock was titrated before and after storing at −80°C to assess the virus titre variation after freezing. The P2 and P3 titres were 1 × 10^7^ PFU/mL and 1 × 10^8^ PFU/mL, respectively.

The analysis of small-scale production allowed to observe a specific trend of cell viability, diameter values, and virus titre depending on different MOIs and times of harvest used.

**Figure 1:**
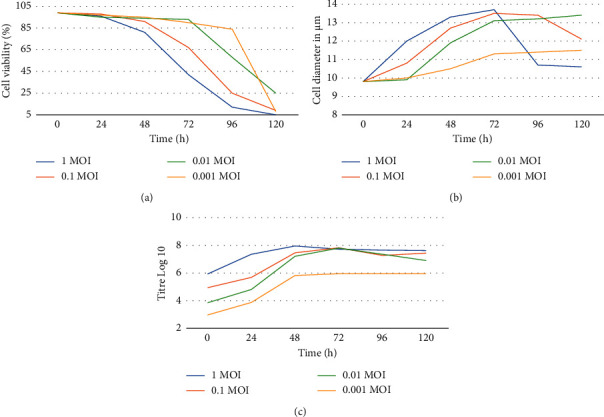
(a) Trend of cell viability (cell/mL) checked over time (0 h, 24 h, 48 h, 72 h, 96 h, and 120 h), after infection of the Sf9 cells with different MOI of recombinant baculovirus. (b) The Sf9 cells diameter (*µ*m) checked at different timepoints (0 h, 24 h, 48 h, 72 h, 96 h, and 120 h) to measure the infection rate of Sf9 by recombinant baculovirus at different MOIs. (c) Trend over time (0 h, 24 h, 48 h, 72 h, 96 h, and 120 h) of the viral titre after infection of the Sf9 cells with different MOIs of recombinant baculovirus.

At the same times of harvest, the virus titre trend in the cells infected at 1, 0.1, and 0.01 MOI was similar except for the cells infected using 0.001 MOI where the titre at 48 h p.i was lower by 2 log10 (Figure 1(a)).

The parameters of cell viability and diameter showed a trend consistent with the concentration of the virus used as inoculum. As shown in Figure 1(b), the cell viability values are inversely proportional to the virus concentration at different time points. Cell diameter (Figure 1(c)) showed an increase until 72 h p.i. and started to decrease reaching the minimum at 120 h, and this trend agrees with the cell viability of Figure 1(b).

Combining the different parameters, the large-scale production was performed using 0.01 MOI and collecting the production at 72 h p.i., and in this condition, the cell viability and diameter parameters were 85% and 13 *µ*m, respectively.

### 3.2. Analysis of Expressed and Purified recVP7 EHDV Protein

A high degree of protein purity was obtained using both purification methods adopted for the pellet and supernatant of infected cells. Significant difference in the quantity of protein obtained from the two different biological sources of the same production was demonstrated using Bradford assay quantification. Indeed, the purification from the pellet of 5 × 10^9^ infected Sf9 cells gave us only about 1 mg recVP7, while we were able to obtain almost 15 mg of recVP7 from the cell supernatant.

The recombinant proteins obtained from the two different purification processes were analysed in SDS-PAGE and Western blotting.

**Figure 2:**
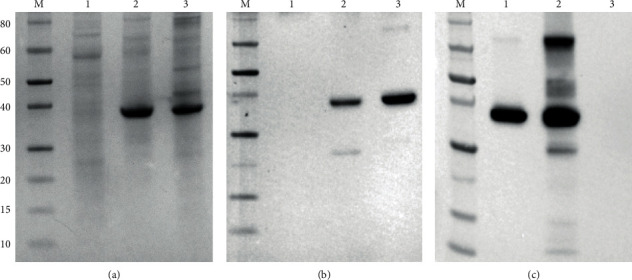
(a) Coomassie staining of SDS-PAGE of recVP7 purified. Lane 1, uninfected Sf9 used as negative control; lane 2, recVP7 purified from the pellet; lane 3, rec VP7 purified from the supernatant. Molecular weight marker, fragment sizes are measured in kDa (M). (b) Western blotting analysis of recVP7 purified, using anti-VP7 mAb clone 4G11 HRP-conjugated. lane 1, uninfected Sf9 used as negative control; lane 2, rec VP7 purified from pellet; lane 3, recVP7 purified from the supernatant. Molecular weight marker, fragment sizes are measured in kDa (M). (c) Western blotting analysis of recVP7 purified using anti-V5 mAb HRP-conjugated. Lane 1, recVP7 purified from the pellet; lane 2, recVP7 purified from the supernatant; lane 3, uninfected Sf9 used as negative control. Molecular weight marker, fragment sizes are measured in kDa (M). All the samples were fractioned by the SDS-PAGE under denaturing conditions.

In Coomassie staining (Figure 2(a)), no specific band was present in the negative control (lane 1); whereas, intense bands, corresponding to the pVP7 and sVP7 recombinant proteins, can be observed at the predicted molecular weight of about 40 kDa (Figure 2(a), lanes 2 and 3).

Figure 2(a) shows a different degree of purity between pVP7 and sVP7; indeed, in lane 2, pVP7 appeared more purified than sVP7 (lane 3).

Purified protein identity was confirmed by Western blotting using a specific anti-VP7 mAb (Figure 2(b)), clone 4G11 (IZSLER), and anti-V5-HRP antibody (Figure 2(c)). The results showed a specific band at molecular weight of about 40 kDa in both samples (Figure 2(b) lanes 2-3; Figure 2(c) lanes 1-2) with both antibodies. No bands were present in the sample corresponding to the negative control (Figure 2(b) lane 1; Figure 2(c) lane 3).

The immunoreaction with mAb 4G11 revealed for the sVP7 sample the presence of a slightly visible band of approximately 78–80 kDa in panel B, the same band was strongly visible after detection using anti-V5 antibody in panel C. The molecular weight of 78–80 kDa corresponds to the dimeric form of recVP7. The samples in lane 2 (pVP7) of panel B and in lane 2 (sVP7) of panel C showed also faint bands of molecular weight lower than 40 kDa, probably due to a slight protein degradation.

### 3.3. Reactivity of recVP7 EHDV in Enzyme-Linked Immunosorbent Assay

**Figure 3:**
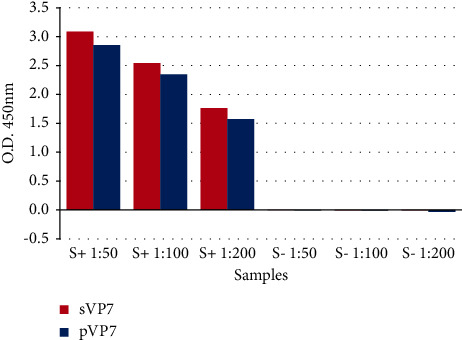
Indirect ELISA: sVP7 and pVP7 used as antigens in i-ELISA to test positive (S+) and negative (S−) sera at different dilutions.

In order to evaluate recVP7 as a diagnostic reagent candidate, both recVP7 purified from pellet and supernatant were used as antigens in i-ELISA at the same conditions (Figure 3).

The results demonstrated that pVP7 and sVP7 had similar reactivity using different dilutions of the positive bovine serum and no reaction with the negative bovine serum used as controls.

As shown in Figure 3, we can observe that sVP7 reached O.D. higher values than pVP7 ones at all dilutions that were used.

**Figure 4:**
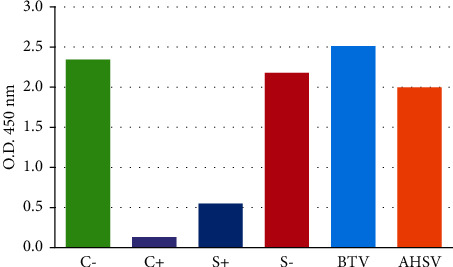
Competitive ELISA using sVP7 as antigen. C−, EHDV-negative serum; C+, undiluted EHDV-positive serum; S+, EHDV-positive camel sera; S−, EHDV-negative camel sera; BTV, BTV-positive sera; AHSV, AHSV-positive sera.

According to the results obtained in i-ELISA, we tested 185 serum dromedary camel samples in competitive ELISA using recombinant sVP7. The analytical specificity of the c-ELISA procedure evaluated using BTV and AHSV-positive sera did not show any cross-reaction (Figure 4).

The O.D. mean values for all the samples tested in new c-ELISA are shown in Figure 4. The positive and negative controls resulted below 0.3 and above 2.5 O.D. values, respectively. The positive dromedary camel sera showed a 0.6 O.D. mean value, whereas the O.D. mean value of negative samples was above 2.2. The method specificity was evaluated testing cross-reactivity against AHSV and BTV-positive sera and showed no reaction; indeed, these samples resulted both negative, showing O.D. mean values of 2 and 2.5, respectively.

**Figure 5:**
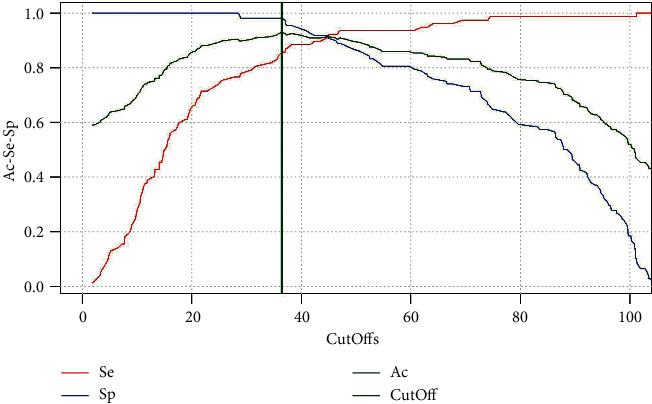
ROC curve for the developed c-ELISA.

Through the analysis of normalized data of the dromedary camel tested sera, it was possible to define a cutoff value of 36.43%. The sensitivity, specificity, and accuracy parameters values of the method were determined at 85.7%, 98.1%, and 93%, respectively (Figure 5).

The results obtained from the comparison between the c-ELISA commercial kit and the new in-house developed test showed an overall agreement; indeed, 66 out of 77 total samples that tested positive with the reference method resulted positive also in our test. Eleven samples that tested positive in the commercial kit were instead negative in our c-ELISA. In the other hand, 106 out of 108 samples that tested negative in the commercial kit were confirmed negative in our test and only 2 samples resulted positive in our method, as given in Table 1.

All the BTV and AHSV positive serum samples tested negative in both assays.

The R software established that the kappa Cohen value is equal to 0.85.

## 4. Discussion

Epizootic haemorrhagic disease is a noncontagious vector-borne disease transmitted by insects of the genus *Culicoides*. The disease is considered endemic in many areas of North America, Australia, and some Asian countries. Currently, no cases of EHDV infection have been notified in Europe, but in the last decade, many cases of EHD have occurred in countries surrounding the Mediterranean basin area, increasing the risk of virus introduction in European continent [11, 15, 35]. The economic impact of EHD is significant considering the direct losses as estimated in previous studies and indirect losses due to animal movement restriction [15, 36].

Therefore, the availability of diagnostic/screening assays can play an important role in surveillance and control programs of the disease, for an early alert from the veterinary official authorities.

In this study, we optimized an efficient recVP7 expression using the baculovirus system, its purification using IMAC, and its application as antigen in a competitive enzyme-linked immunoassay (c-ELISA).

To develop an efficient recombinant protein production, we tested several experimental conditions monitoring different parameters such as cell viability, cell diameter, and viral titre using different MOIs and TOHs. Large-scale protein production was obtained using 0.01 MOI and harvesting cells at 72 h p.i. These conditions allowed us to obtain a high quantity of protein with a high degree of purity.

Eventhough efficient production of EHDV VP7 using a baculovirus system had already been reported [37, 38], in this study, we describe for the first time a successful purification of the protein from cell culture supernatant.

The use of an automated system to purify recVP7 through affinity chromatography offered several advantages, speeding up the process, and its standardization [39–42]. We tested several conditions, and the highest quantity of purified recVP7 was obtained from the supernatant; indeed, the amount of VP7 purified from the supernatant was 15 mg, in contrast to 1 mg obtained from the pellet, even if the protein purity level of sVP7 was lower than the purity of pVP7, as already occurred in previous studies [33].

The purified sVP7 and pVP7 were characterized in immunoblotting and subsequently evaluated as reagents in i-ELISA. The two proteins showed similar reactivity in i-ELISA; thus, considering the high quantity and the easy one-step purification procedure of sVP7, we used this fraction to develop c-ELISA. The peroxidase-conjugated mAb 4G11 (IZSLER) was employed with the protein as the detector and competitor system to evaluate 185 field sera of camelids from UAE, Sudan, and Pakistan including samples from EHDV naturally infected animals.

The developed assay showed optimal values of sensitivity (85.7%), specificity (98.1%), a high accuracy (93%), and an excellent coefficient of agreement comparing the results with the only diagnostic commercial kit available. We used it as the reference method. Both methods gave concordant results in the most samples analysed. Because of the small sample amount, we could not further analyse the 13 discordant samples using the gold standard method (virus neutralization test) to determine the result trueness.

Viruses belonging to *Orbivirus* genus possess several morphological and structural similarities that have been causing significant difficulties in the disease differential diagnosis. In this study, we were able to develop a test that showed a good discriminatory capacity between EHDV, BTV, and AHSV. Positive sera against these viruses were tested in our c-ELISA, and the results showed a high specificity of the method recognising them as negative for EHDV.

The good assay performance is associated to the use of a specific mAb and to the use of a purified recombinant protein. Indeed, previous studies [39, 43] reported that unpurified VP7 antigen is usually associated with less stability than purified antigen. The presence of extraneous proteins with enzymatic activity or the intrinsic characteristic of viral proteins to assemble into virus-like structures can cause an uncontrolled aggregation of the proteins within the sample. Some authors reported the use of unpurified recombinant antigen [37, 38] and purified recombinant antigen from pellet [39] in c-ELISA. Even if in these studies the test has had good performances, the protein obtained in this study was purified and therefore more stable than the unpurified antigens. Furthermore, the purifying process adopted was developed from supernatant and is less laborious than the procedure reported by others authors [38, 39].

The standardization of the purifying process and the high level of purifications of the antigen have a key role to set up an assay for large-scale production, screening, and diagnostic purposes in endemic areas.

In the last decade, several studies reported the presence of camels' antibodies against different viral emerging diseases, such as bluetongue, peste des petits ruminants, West Nile disease, African horse sickness, epizootic haemorrhagic disease, and Rift Valley fever [11, 35, 44–46].

In particular, some studies revealed the presence of EHDV antibodies in camels demonstrating the susceptibility of this animal species to the virus [17, 18].

In this study, in order to evaluate the performances of our c-ELISA, we tested the same serum samples that Wernery and colleagues (2013) used to investigate the presence of antibodies against EHDV in dromedary camels.

To date, the only commercially available kit used in this study was validated for sheep, goat, cattle buffalo, and deer species; thus, the necessity of a specific test for camels arises from the growing diagnostic need for EHDV identification. Indeed, camels have been used as sentinel animals in monitoring the spread of several emerging diseases, included EHD [35, 44].

Even if the data showed in this work cannot be considered a validation of our method, the results obtained testing dromedary camel sera allowed us to affirm that our c-ELISA could be used to detect EHDV antibodies in camels. The c-ELISA based method developed in this study provide, for the first time, a rapid serological test specific for camel species and represent not only a diagnostic assay but an important instrument to monitor the dissemination of the disease and prevent the risk of insurgence in nonendemic countries. Further investigation could be performed in order to understand the potential application of our test to the species other than camels.

## Figures and Tables

**Table 1:** Results of positive and negative sera tested using new c-ELISA and commercial c-ELISA.

	Commercial kit (ID screen EHDV competition)		
c-ELISA (IZSAM)	Positive	Negative	Total
Positive	66	2	68
Negative	11	106	117
Total	77	108	185

## Data Availability

Data are available from the corresponding author upon request.
